# Sex-dependent gene expression in early brain development of chicken embryos

**DOI:** 10.1186/1471-2202-7-12

**Published:** 2006-02-15

**Authors:** Birger Scholz, Kim Kultima, Anna Mattsson, Jeanette Axelsson, Björn Brunström, Krister Halldin, Michael Stigson, Lennart Dencker

**Affiliations:** 1 Department of Pharmaceutical Biosciences, Division of Toxicology, The Biomedical Center, Husargatan 3, Box 594, SE-75124 Uppsala, and Centre for Reproductive Biology in Uppsala, Uppsala University, Sweden; 2 Department of Environmental Toxicology, Uppsala University, Norbyvägen 18A, SE-75236 Uppsala, and Centre for Reproductive Biology in Uppsala, Uppsala University, Sweden; 3 Institute of Environmental Medicine, Karolinska Institutet, P.O. Box 210, SE-171 77 Stockholm, Sweden

## Abstract

**Background:**

Differentiation of the brain during development leads to sexually dimorphic adult reproductive behavior and other neural sex dimorphisms. Genetic mechanisms independent of steroid hormones produced by the gonads have recently been suggested to partly explain these dimorphisms.

**Results:**

Using cDNA microarrays and real-time PCR we found gene expression differences between the male and female embryonic brain (or whole head) that may be independent of morphological differentiation of the gonads. Genes located on the sex chromosomes (ZZ in males and ZW in females) were common among the differentially expressed genes, several of which (*WPKCI-8*, *HINT*, *MHM non-coding RNA*) have previously been implicated in avian sex determination. A majority of the identified genes were more highly expressed in males. Three of these genes (*CDK7*, *CCNH *and *BTF2-P44*) encode subunits of the transcription factor IIH complex, indicating a role for this complex in neuronal differentiation.

**Conclusion:**

In conclusion, this study provides novel insights into sexually dimorphic gene expression in the embryonic chicken brain and its possible involvement in sex differentiation of the nervous system in birds.

## Background

Sexual differences that arise in the brain during embryonic development underlie sex-specific reproductive behavior in adults of vertebrate species. Better knowledge of the mechanisms behind sexual differentiation of the nervous system can contribute to the understanding of the brain's functions and its susceptibility to disease [1], and is critically needed in the context of developmental neurotoxicity and chemically induced disruption of the neuroendocrine system [2]. For a long time the idea prevailed that somatic tissues in the embryo are gender neutral until the gonads become hormonally active following their sex-specific differentiation, a process presumed to be governed by cell-autonomous programs genetically determined by the sex chromosomes [3-5]. However, several recent studies indicate that sex-specific neuronal characteristics may be influenced by genetic mechanisms preceding or occurring in parallel with the effects produced by the gonadal hormones [6]. That the genetic sex of neurons contributes to the process of sexual differentiation is illustrated by studies of the neural song circuit in a gynandromorphic zebra finch, where genetically male and female brain cells develop differently in the presence of the same levels of circulating gonadal hormones [7].

Birds, in which males are homogametic (ZZ) and females heterogametic (ZW), provide attractive models for the study of normal and disturbed sex-related neurobehavioral development [7,8]. The recent advent of chicken expressed sequence tag (EST) clone collections [9,10] and the release of the first draft sequence of the chicken genome [11] has paved the way for genome-wide studies of sexual differentiation in the chicken. In the chicken, the indifferent embryonic gonad rudiments differentiate morphologically around embryonic day (ed) 6.5 [12,13], whereas the expression of enzymes involved in steroidogenesis can be detected as early as ed2 [12], and estrogen receptor (ER) mRNA is detectable in the male and female urogenital system from ed4.5 [14]. The earliest signs of sexually dimorphic expression of genes related to gonadal hormones and steroidogenesis, however, have been detected at ed5 when mRNA for anti-Müllerian-hormone is more abundant in males [15], and aromatase [16] in females.

In the present study, we used cDNA microarrays to investigate if there are gene expression differences between the male and female embryonic brain (or whole head) that may be independent of morphological differentiation of the gonads. We report evidence for sex-related gene expression differences from ed4 (whole head) through ed10 (brain) for several genes located on sex chromosomes. We propose that intrinsic genetic mechanisms are involved in sexual differentiation of the chicken brain, and that sex-linked genes may play key roles.

## Results

### Identification of gene expression differences between male and female embryonic chicken brains

**Figure 1:**
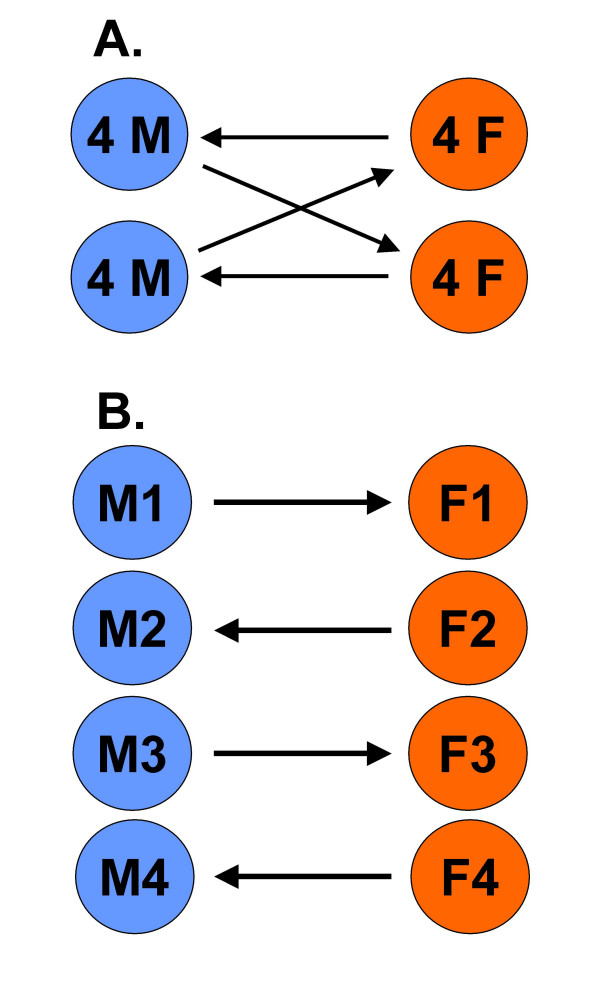
Experimental design. A total of 20 microarray hybridizations were performed, addressing biological variation through the use of 12 biologically independent samples for each sex, and technical variation through Cy3/Cy5 dye reversal as follows. (A) For each of the four embryonic stages (ed4, 6, 8 and 10), two samples per sex were generated by pooling equal amounts of total RNA from four individual male (M) or female (F) whole heads (ed4 and 6) or brains (ed8 and 10). Each RNA pool was converted to two cDNA samples, tagged with Cy3 and Cy5-capture sequence (Genisphere), respectively, and hybridized to two microarray slides, as illustrated by arrows. Arrowhead indicates Cy5-labeled sample. (B) For ed10, total RNA from four individual male (M) and female (F) brains were separately tagged with either Cy3 or Cy5-capture sequence during cDNA synthesis, and analyzed on four microarray hybridizations with dye-reversal as illustrated by arrows.

**Figure 2:**
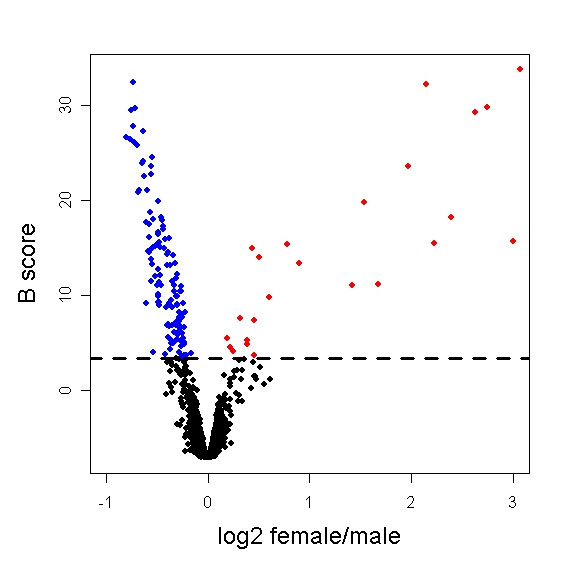
Microarray analysis of gene expression differences between female and male chicken embryos across four embryonic stages (ed4, 6, 8 and 10) in the whole head (ed4 and 6) and brain (ed8 and 10). The volcano plot shows the magnitude of differential expression (log_2 _female/male ratio) versus the posterior log odds of differential expression (B score) for each of 10,702 microarray clones (each represented by a dot) included in the analysis (see Methods). The horizontal line marks the threshold (B score > 3.413) for selecting a clone as significantly (adjusted p-value < 0.0005) more highly expressed in males (blue) or females (red).

**Figure 3:**
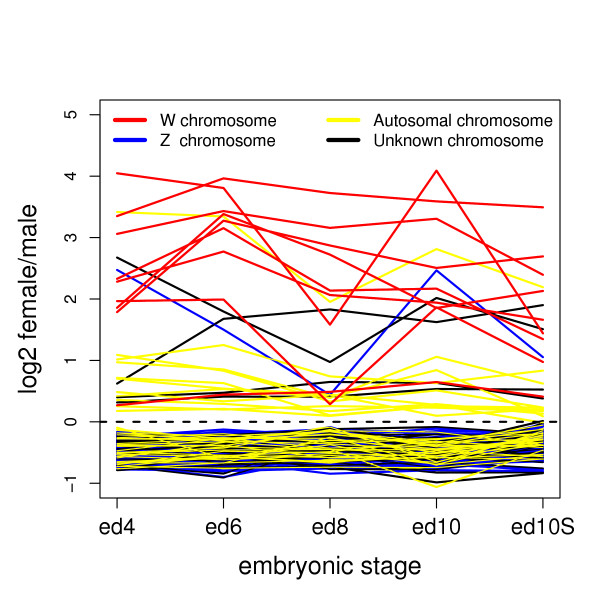
Expression difference between females and males of W-linked (red), Z-linked (blue), and autosomal (yellow) genes, and genes with unknown location (black), at ed4, 6, 8 and 10, respectively, for the 146 clones identified as significantly differentially expressed (adjusted p-value < 0.0005) in the microarray analysis. The log_2 _female/male ratios are averages between four replicate microarrays, using either the design illustrated in Figure 1A (ed4, ed6, ed8 and ed10), based on pools, or the design in Figure 1B (ed10S), based on separate individuals.

To study sex-specific gene expression in the developing brain of chicken embryos during stages around morphological differentiation of the gonads, total RNA extracted from male and female heads on ed4 and ed6, and brains on ed8 and ed10, was subjected to replicated microarray analysis using cDNA microarrays containing approximately 14,000 EST clones. For each sex, we generated a total of 12 biologically independent samples, comprising either individuals (ed10) or pools of four individuals (ed4, 6, 8 and 10). These 24 samples were analyzed on 20 array slides, with technical replication as shown in Figure 1. Using an empirical Bayes moderated t-test [17], and adjusted p-values according to Benjamini and Hochberg [18], we found 146 clones with significant differential expression (adjusted p-value < 0.0005, corresponding to a B score threshold of 3.413) across all investigated embryonic stages (Figure 2). For most of these clones, the log_2 _female/male ratios were relatively similar across the studied developmental stages (Figure 3, see Additional File 1). Importantly, for a majority of these clones the expression differences between males and females were present at the earliest embryonic stage (Figure 3, see Additional File 1), i.e. well before morphological gonadal differentiation.

### Characterization of differentially expressed genes

The 146 identified clones (see Additional File 2) were found to represent 133 genes, 19 of which were more highly expressed in females, and 114 in males (Table 1). Among the 'female genes', three are located on the female-specific W chromosome (Table 2), one on the Z chromosome (Table 2) and 11 on autosomes, whereas for four genes the location is unknown (Table 1). Among the 114 'male genes', 61 are located on the Z chromosome (Table 2) and 29 on autosomes, and for another 29 genes the location is unknown (Table 1). Hence, given the fraction of Z-linked clones present in the microarray (257 clones out of ~14,000), genes annotated as being located on the Z chromosome appeared overrepresented among the differentially expressed genes (Table 1). As the chicken W chromosome is still poorly characterized, with many gaps in the sequence, the same conclusion is less straightforward to draw about W-linked genes (for further details see the UCSC Genome Browser Gateway [19]).

The differential expression of selected genes on ed4 (and, for some genes, ed8) was investigated by real-time PCR of individual samples (see Methods) and the results (see Additional File 3) are presented below in the context of the chromosomal locations of these genes.

### W-linked genes

Since the W chromosome is present only in female chickens, the expression of W-linked genes was reflected in the microarray analysis by high log_2 _female/male ratios (Figure 3), and in the real-time PCR analysis by even higher (technically 'infinite') ratios (Table 3). An exception from this was *SPINW*, for which the exclusive expression in females was not indicated by similarly high log_2 _female/male ratios in the microarray analysis (see Additional File 2). However, unlike what is known for the other W-linked genes reported here, *SPINW *has a counterpart (*SPINZ*) on the Z chromosome (see below). *SPINW *was found by real-time PCR to be expressed in the female head on ed4 (Table 3, see Additional File 3). Also the expression of *WPKCI-8*, for which female expression at all stages was detected by seven clones in the microarray analysis (see Additional File 2), was verified in the ed4 female head by real-time PCR.

**Figure 4:**
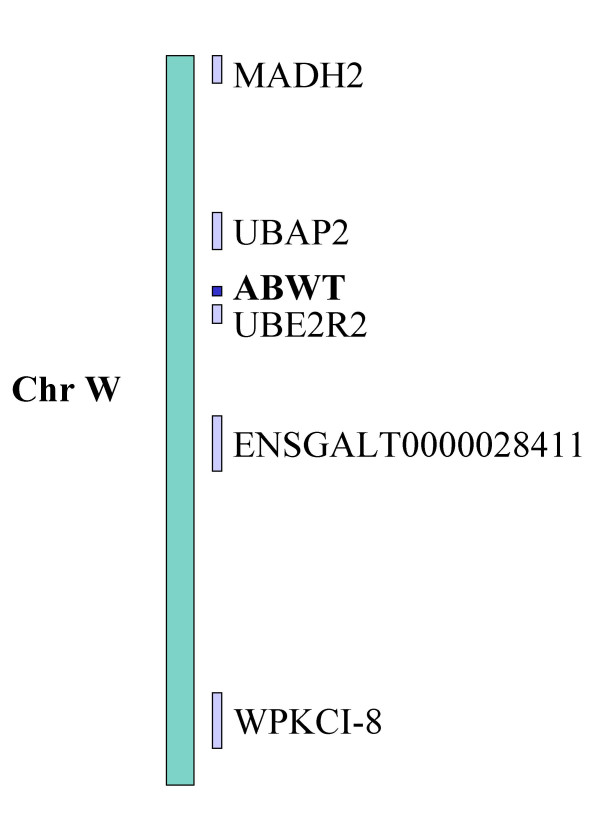
Relative location of Avian brain W-linked transcript (*ABWT*) in the W chromosome. The sequence of EST clone WLA084D05 (*ABWT*) aligns to the chicken W chromosome adjacent (~300 nt) to the gene encoding the chicken candidate for ubiquitin-conjugating enzyme E2R2 (*UBE2R2*). Information for the relative locations was retrieved from the UCSC Genome browser [19] and encompasses the region in chrW_random:1-455,598 (also see Additional File 3).

The sequence of the EST clone WLA084D05 shows no similarity to any known gene or putative protein. It is part of the UniGene entry Gga.16155 and the TIGR Gene Index entry TC216880 (Release 10.0), located about 300 nucleotides from a chicken sequence similar (>90% sequence alignment match at the amino acid sequence level) to the *Ube2r2 *mouse sequence (see Additional Files 4 and 5). Using primers designed to amplify this putative *Ube2r2 *homolog, we did not detect any significant differential expression in embryonic chicken heads. However, using primers amplifying the WLA084D05 sequence, we found expression in heads from 4-day-old female embryos (Table 3). It remains to determine whether this EST may represent a novel W-linked gene (Figure 4, see Additional File 4), which we provisionally suggest calling 'avian brain W-linked transcript' (*ABWT*).

### Z-linked genes

**Figure 5:**
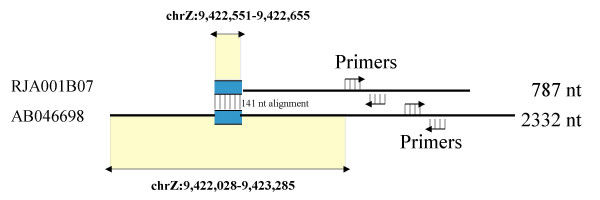
Comparison of MHM transcripts. Alignment between the sequence of the EST clone RJA001B07 (787 nt) and the Z-linked MHM-region sequence AB046698 (2332 nt), showing a 141-nt region with 98% sequence similarity (blue boxes). The yellow fields are the regions of BLAT alignment to the Z-chromosome (RJA001B07: chrZ:9,422,551-9,422,655 and AB046698: chrZ:9,422,028-9,423,285) in the first assembly of the chicken genome [19]. Also indicated are the approximate locations of the two primer pairs used in the real-time PCR to confirm the higher expression in females of each MHM transcript.

Whereas half of the genes (56 of 114) suggested from the microarray study to be more highly expressed in males are located on the Z chromosome, only one clone with female-enhanced expression is known to be Z-linked (Tables 1 and 2). We found 141 nucleotides of this clone (RJA001B07) to align with high similarity (98% nucleotide sequence identity) to a partial sequence of the GenBank entry with accession AB046698 (Figure 5), representing a Z-linked male hypermethylated (MHM) region. This sequence is thought to be specifically transcribed into a non-coding nuclear RNA in females [20]. Using primers designed to discriminate between the RJA001B07 and AB046698 sequences (Figure 5), we found female-specific transcription for both of these MHM genomic regions (Table 4).

Real-time PCR analysis confirmed the male-enhanced microarray expression for all Z-linked genes examined (Table 4). We also found significantly higher expression in males (p < 0.05) for two Z-linked genes (*CDK7 *and the Z-linked *WPKCI-8 *homolog *HINT*), but not for a third (*DMRT1*), that were not represented in the microarray but are functionally related (see Discussion) to other genes identified as differentially expressed (Table 4).

A close to two-fold higher expression in males was observed for most of the Z-linked genes in the real-time PCR analysis (Table 4), suggestive of gene dosage. Exceptions were *BTF2-P44*, *CDK7 *and *HMGCOA*. No differential expression was detected by real-time PCR for three genes (*ANXA1*, *ZO-2 *and *ZOV3*) that were randomly selected among the non-differentially expressed Z-linked genes in the microarray experiment (Table 4), supporting that only certain Z-linked genes are more highly expressed in males. A fourth Z-linked gene (*TXN*) randomly selected among genes that were not differentially expressed in the microarray analysis was revealed by real-time PCR to be more highly expressed in males (Table 4). However, examination of the corresponding microarray clone (RJA044E12) by sequencing showed that the spotted cDNA contained vector sequence only.

### Genes located on autosomes or having unknown chromosomal location

Differential expression could be confirmed by real-time PCR for only three genes out of 14 analyzed genes with autosomal (*P311*) or unknown location (*ACAA2 *and *CETN3*), all with higher expression in males (Table 5). Errors in the microarray were uncovered for 6 of 11 genes that could not be confirmed by real-time PCR (Tables 4 and 5). For example, the microarray spots thought to represent the male-enhanced autosomal genes *AATF *and *ACAD8 *were found to contain EST clones representing the Z-linked genes *RPS6 *and *PLAA*, respectively (Table 5). Two of the microarray clones showing female-enhanced expression that could not be confirmed by real-time PCR were found to be erroneous and contain repetitive sequences (data not shown), whereas two such clones (representing the autosomal genes *CEZANNE2 *and *NAT5*) appeared to be correctly annotated (Table 5). The discrepancy between microarray and real-time PCR data for non-sex chromosomal gene expression remains unexplained. Unspecific cross-hybridization of sex chromosome-linked target cDNA to non-sex chromosomal array probes may be one possibility. Because of this uncertainty we decided not to further follow up on non-sex chromosomal candidates. Only microarray data on sex-chromosomal genes are reported in Table 2 (for the whole data set of 146 clones, see Additional File 2). We confirmed the absence of differential expression for the autosomal gene *MAT1*, a gene selected for PCR analysis because of its functional association with other genes identified as differentially expressed (see Discussion).

## Discussion

Relatively little is known about the genes that are involved in neuronal sex differentiation in birds, whether influenced by gonadal steroid hormones or not. In the present study we used cDNA microarrays to identify genes that are differentially expressed between males and females in the developing chicken brain (or whole head) before (ed4) and during (ed6, 8 and 10) morphological differentiation of the gonads. We found statistical significance (adjusted p-value < 0.0005) for differential expression of 146 microarray clones across the embryonic stages and types of tissue sample (whole head for ed4 and 6, and brain for ed8 and 10). These clones correspond to ~1.4% of the ~10,700 clones included in the analysis. Using real-time PCR we were able to confirm female expression for all three W-linked genes identified, and the differential expression of all ten Z-linked genes tested and three genes with autosomal or unknown location. The real-time PCR analysis furthermore revealed one false negative among Z-linked genes (TXN), and some false positives among genes located on autosomal or unknown chromosomes. In many cases, however, this could be traced to errors in the microarrays.

A major finding was that sex-specific differential expression could be detected for numerous genes already at the earliest studied embryonic stage (ed4), before the stage (~ed5) at which the earliest sex differences in gonadal gene expression with regard to steroidogenesis have been found [15,16,21]. Expression of several genes for steroidogenic enzymes (P450scc, P450c17, 3-βHSD, 17βHSD, and aromatase) in chicken gonads already on ed4 has been documented, but no sex-dependent expression differences [21]. Even if we cannot rule out the possibility that hormonal differences between males and females may exist on ed4, the early sex-specific expression of the genes reported in the present study may be primarily regulated by other mechanisms than hormonal control. In favor of this notion, we found no apparent relationship between the expected differences in hormonal levels associated with gonadal differentiation [12] and the magnitude of the expression differences between the sexes for these genes (Figure 3). Moreover, our preliminary data from chicken embryos exposed to ethinyl estradiol *in ovo* on ed4 show that the expression in the brain on ed10   for the majority of these genes is virtually unaffected by treatment in either sex (work in progress).

Gene expression differences independent of gonadal hormones have previously been suggested to play critical roles in the sexual differentiation of the brain in birds [22] and mice [23]. Why an apparently different set of genes should be involved in the chicken compared to the mouse [23] is unclear. That such a large proportion of genes located on the sex chromosomes (Z and W) were identified as differentially expressed in the chicken is therefore intriguing considering the independent evolution and different gene content of the sex chromosomes in birds and mammals [24].

A majority of the genes indicated to be differentially expressed from the microarray analysis were more highly expressed in males than in females, and about half of these genes are located on the Z chromosome. This raises the issue of dosage compensation versus biallelic expression of Z-linked genes in males [25]. Although biallelic expression has previously been demonstrated [26,27], and might be inferred from the close to two-fold higher expression in males of many Z-linked genes in our study, the expression levels of some Z-linked genes were found both by microarray and PCR analysis not to differ significantly between the sexes. Such a lack of sex differences in the expression of certain genes located on the Z chromosome would be in line with previous evidence for dosage compensation [28]. Taken together, our results suggest that if dosage compensation does occur in chicken it is unlikely to involve a widespread inactivation of the Z chromosome similar to that of the mammalian X chromosome. Studies by McQueen and coworkers [28] in chicken embryos have shown that at least six genes on the Z chromosome are dosage compensated. However, Kuroda and coworkers [26] found that transcription of five genes, including two of the genes studied by McQueen and coworkers [28], is taking place on both Z chromosomes of male chicken. This provides further support that dosage compensation in birds does not involve inactivation of a large majority of genes on the Z chromosome.

That epigenetic mechanisms such as methylation and acetylation may be involved in the sex-specific expression of genes located on the Z chromosome has previously been implicated by the female-specific expression of the Z-linked MHM region [20,29]. In agreement with this, our microarray results showed high female/male ratios suggestive of expression in females only of two distinctive transcripts containing an MHM region. Whether these transcripts, represented by microarray clone RJA001B0 (Table 2) and GenBank entry AB046698[20], respectively, are expressed from the same or independent genomic regions is unclear. Their highly similar MHM regions (141 nucleotides with 98% identity) and their female-specific expression indicate some common function, possibly the repression of the adjacent gene *DMRT1*, suggested as a conserved sex determining gene [30-35], through the accumulation of a non-coding RNA [20]. However, unlike in male gonads before and during gonadal differentiation [32,36,37], we found no evidence of male-enhanced expression of *DMRT1 *in the embryonic chicken brain.

**Figure 6:**
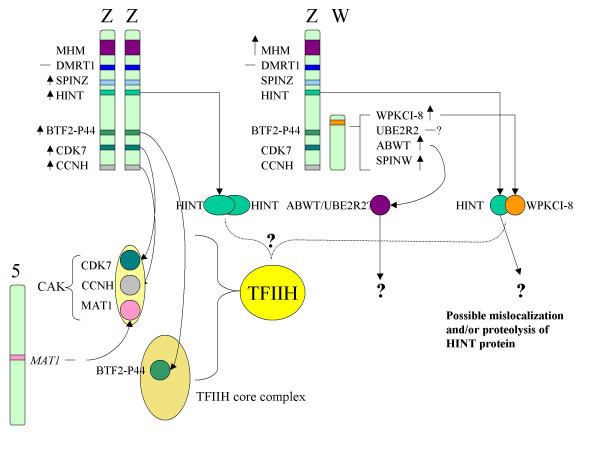
Differentially expressed genes related to sex determination/differentiation and/or the TFIIH protein complex (see Discussion). Higher expression in males (ZZ) or females (ZW) of Z-linked and W-linked genes is indicated by arrows pointing upward. Short arrows () indicate ≤2-fold difference, and long arrows (↑) indicate >2-fold difference, as determined by real-time PCR. Genes not differentially expressed are indicated with a dash (—). Long thin arrows (→) indicate gene product associations. A dotted bracket shows the possible interaction between HINT and the TFIIH complex.

We found high female/male expression ratios on ed4 for three genes/clones (*WPKCI-8*, *SPINW *and *ABWT*) located on the female-specific W chromosome, indicating significant early expression in the female embryonic brain. Because of its early expression (ed4.5) in the developing female gonads [38,39], *WPKCI-8 *(also known as *ASW*) has been suggested to be involved in avian sex determination [13]. Early *WPKCI-8 *expression (ed5) has also been detected in the spinal cord, spinal ganglion, and myotomes [38]. The *WPKCI-8 *gene, which is reiterated approximately 40 times on the W chromosome [25], and its single-copy homolog on the Z chromosome *HINT *[25] both encode proteins belonging to the Hint family of dimeric nucleotide hydrolases [38]. The Wpkci-8 protein, however, lacks the histidine triad (HIT) motif [25] and appears to act as a dominant suppressor of Hint activity, possibly by conferring signals for mislocalization or degradation of the Hint/Wpkci-8 heterodimers [40] (Figure 6). It is an interesting possibility that suppressed levels of active Hint in the brain of female chicken embryos, resulting from both lower expression of the *HINT *gene itself and inhibition by expressed Wpkci-8, could contribute to sexual differentiation of the nervous system. Female expression of *WPKCI-8 *and male-enhanced expression of *HINT *has previously been detected in the telencephalon of juvenile zebra finches [41]. The Hint protein (also known as protein kinase C inhibitor/interacting protein) is well conserved between species [38,39], but whether this protein is involved in sex determination or sexual differentiation in various species is unclear.

We found male-enhanced expression for two Z-linked genes (*CCNH *and *CDK7*) known to encode subunits of the cyclin-dependent kinase activating kinase (CAK) complex, and a third one (*BTF2-P44*) encoding a subunit of the transcription factor II H (TFIIH) core complex. Together these complexes constitute the basal transcription complex TFIIH, which is involved in transcription, DNA repair, and cell-cycle control [33]. Among the genes encoding the nine proteins (CDK7, CCNH, MAT1, ERCC2, ERCC3, BTF2-P34, BTF2-44, BTF2-52, BTF2-62) in this complex [33], two (ERCC2 and BTF2-52) appear to be less conserved in the chicken genome (see Additional Files 6 and 7). The implications of the male-enhanced expression of some but not all components of CAK (we found no differential expression for its third component MAT1) and the TFIIH core complex remains to be elucidated. However, it is intriguing that both CAK and TFIIH are involved in estrogen receptor α (ERα) and androgen receptor (AR) transactivation in mammals [42-45], and that a functional CAK is important for both the mitotic and meiotic cell cycle [46-48]. The proteins encoded by the Z-linked genes *HINT *and *APTX*, are both members of the HIT protein superfamily [49]. *APTX*, which in the microarrays is more highly expressed in males, encodes aprataxin, a protein involved in single-strand break repair [50,51] and Hint seems to function as a positive regulator of components of TFIIH [40], including the *CCNH *homolog Ccl1 [52]. Considering the role of TFIIH in DNA-repair, a sex specific control of DNA repair seems possible. In addition to TFIIH components, three other Z-linked genes associated with DNA repair (*RAD1 *[53], *MSH3 *[54], and *DHFR *[55]) were found in the microarray result to be more highly expressed in males.

Analogous to the sex-dependent expression of *WPKCI-8 *and *HINT*, the W-linked gene *SPINW *was expressed in females while the Z-linked homolog *SPINZ *showed male-enhanced expression. *SPINW*, which is prominently expressed in ovarian and somatic tissues, has been suggested to be involved in gonadal differentiation [56], but the early (ed4) sex-specific expression of these genes in the brain may indicate a more widespread involvement in sexual differentiation. We also identified a W-linked transcript, *ABWT*, which in addition to being expressed in the early female embryonic brain has also been found in cDNA libraries from chicken ovaries and heart (UniGene entry Gga.16155). *ABWT *maps to the genome in the vicinity of the homolog to mouse *Ube2r2 *(*i.e*. chicken gene *UBE2R2*), a gene belonging to the E2 family of ubiquitin-conjugating enzymes [57]. As we were unable to detect any differential expression of *UBE2R2 *transcripts, however, the detected *ABWT *transcripts appear to be distinct from these. It remains to be determined whether *ABWT *is a splice variant of a *UBE2R2 *transcript, a novel gene encoding a yet unidentified protein or a non-coding RNA.

## Conclusion

In conclusion, this study provides hints to mechanisms behind sexual differentiation of the nervous system in birds and raises several new questions. The expression of W-linked genes supports the presence of 'sex-control genes' similar to the mammalian SRY gene, in addition to the possible gene dosage effects from genes on the Z chromosomes. Future studies will reveal if the W- and Z-linked genes are differentially expressed in the early embryonic brain of different bird species and if differential expression of these genes is present also in other tissues. It should be noted, however, that *WPKCI-8*, an apparent key player in sexual differentiation [13,38], is not reiterated on [38], and may even not be linked to [39], the W chromosome of the primitive ratites (emus and ostriches). The crucial importance of sex steroids for organization of the brain and behavior in birds has been shown in several studies (as reviewed by Balthazart et al [58] and Panzica et al [59]), but the results presented in this paper point toward the possible presence also of a genetic component in sex-specific neuronal differentiation. Consequently, it seems likely that genetic and hormonal control interact to organize the avian brain dimorphism during differentiation.

## Methods

### Embryos and sample collection

Fertilized eggs from White Leghorn fowl were purchased from OVA Production (Morgongåva, Sweden). The eggs were incubated at 37.5°C and 60% relative humidity, and were turned every 3 hours. After 8 or 10 days of incubation (ed8 and ed10), whole brains were dissected out. As brains could not be excised in a reproducible way from earlier embryos (ed4 and ed6), whole heads were collected. Heads were excised along a line from the mandibular and maxillary processes of the first pharyngeal arch to the caudal boundary of the myelencephalon (ed4), or from immediately in front of the maxillary process to the caudal boundary of the myelencephalon (ed6). Samples were immediately frozen in liquid nitrogen, and stored at -70°C. A tissue sample from each embryo was also collected for DNA isolation and genetic sexing according to a PCR-based method [60] in which intron sequences of different lengths in the W-linked gene *CHD1W *(females) and Z-linked gene *CHD1Z *(both sexes) are amplified.

### RNA isolation

Total RNA was isolated using the Micro-to-Midi Total RNA Purification System (Invitrogen, Carlsbad, CA). Any contaminating genomic DNA was digested by DNase treatment (DNA-free, Ambion, Austin, USA), according to the manufacturer's recommendations. RNA quality was checked using the Agilent 2100 Bioanalyzer and the RNA 6000 LabChip (Agilent Technologies, Palo Alto, CA, USA). Only high quality RNA, with no signs of degradation, was used for further experiments.

### Microarrays

Spotted cDNA microarrays, containing 1,136 expressed-sequence-tag (EST) clones from a cDNA library of developing chicken brain [9] and 12,771 EST clones from four cDNA libraries from brain and testis of White Leghorn and Red Jungle Fowl [10], spotted in duplicate, were purchased from the Royal Institute of Technology, Stockholm, Sweden [61]. Annotation and chromosome localization of the spotted clones can be found at the Stockholm Bioinformatics Center (SBC) [62].

### Experimental design

Four microarray hybridizations were done for each embryonic stage (ed4, 6, 8 and 10), addressing biological and technical variation through a pooling and dye reversal strategy (Figure 1A). Equal amounts of total RNA extracted from eight male and eight female individual embryos were mixed in four pools with RNA from 4 embryos of the same sex in each pool.

This design, which we adopted from Churchill [63], is a trade-off between the need for biological and technical replication while keeping down animal consumption and microarray use. Although pooling is intended to average between individuals there is a risk that single individuals may bias the sample. Individual variation among the embryos included in the pools was addressed in the PCR analysis (see below), and was found to be relatively modest for the randomly selected individuals assayed (see Additional File 3). Moreover, four additional microarray hybridizations were done for ed10, using an alternative design (Figure 1B) in which all samples were derived from individual embryos and using other embryos than in the pooling strategy.

### cDNA synthesis and microarray hybridization

Labeling of cDNA for microarray hybridization was done using the 3DNA Array 350 Expression Array Detection Kit (Genisphere Inc., Hatfield, PA) according to the manufacturer's protocol and recommendations. Briefly, total RNA (20 μg) was oligo-dT-primed with Cy3- or Cy5-capture-sequence primer (see Figure 1 for dye reversal scheme), and reverse transcribed using SuperScript II Reverse Transcriptase (Invitrogen, Carlsbad, CA). For cDNA hybridization, 5 μg cDNA was mixed with 1 μl mouse COT-1 DNA and either regular (ed8 and ed10S) or Enhanced (ed4, ed6, ed10) hybridization buffer. After hybridization at 60°C for 16 hours, microarray slides were washed on a rocking platform, first with 2 × SSC containing 0.2% SDS for 10 min at 55°C, followed by 2 × SSC and 0.2 × SSC for 10 min each at room temperature. 3DNA hybridization was performed at 60°C for 2.5 hours, followed by washes as for the cDNA hybridization.

### Microarray data analysis

The microarrays were scanned with a GenePix 4000B scanner (Axon, Foster City, CA) at 10 μm resolution. The photomultiplier tube voltage settings were varied to obtain maximum signal intensities while saturating less than 0.1% of the spots. Images were analyzed with the GenePix Pro 5.0 (Axon) software, utilizing the option to find irregular features. Spots with visible artifacts or containing fewer than 35 pixels were manually flagged as bad. As each array contained the same clone spotted in duplicate in each half of the array, the average of the two duplicate spots was calculated for each array. If one of the duplicate spots was missing or flagged bad, the value was based only on the remaining spot. The base 2 logarithm (log_2_) ratio of the median spot intensity for each channel was used to quantify the fold difference in relative gene expression levels. Since all arrays had low background, no background subtraction was done. To remove systematic sources of variation, within-print-group loess normalization [64] was done, in which the relative weight of 0.1 was given to spots flagged as missing or bad. To identify clones differentially expressed between females and males independently of embryonic stage (ed4, 6, 8 or 10), tissue origin (brain or whole head), or experimental design (pools or individuals; see above), the arrays were collapsed across these parameters, resulting in 12 biologically independent samples per gender analyzed on 20 arrays.

Clones for which values were obtained for less than 17 (out of 20) arrays were excluded from further analysis, resulting in 10,792 clones across all arrays. In addition, 90 of the microarray clones known from previous RepeatMasker analysis to mainly consist of repetitive sequence were excluded from further analysis. The moderated t-statistics and log-odds of differential expression was calculated using the Limma package version 2.0.4 [17] in the statistical software R version 2.2.1 [65] (both freely available). To address the problem with multiple testing, the p-values were adjusted using the method of Benjamini and Hochberg [18]. Because of the technical replication of the pooled samples (Figure 1A), only 12 arrays (not all 20) were independent measurements. We only considered clones to be differentially expressed if the adjusted p-value < 0.0005. Microarray data are available in the ArrayExpress database [66] at the accession number E-MEXP-266.

### Annotation of EST clones detecting differential expression

We reevaluated gene identities for all differentially expressed EST clones (146 clones; see Results) by searching The Institute of Genomic Research (TIGR) Chicken Gene Indices (Release 10.0) [67]. All sequences can be accessed by using the clone identity in a search against GenBank database [68]. Functional annotations were reassessed by RepeatMasker [69] and searching the National Center for Biotechnology Information (NCBI) non-redundant and the Universal Protein Resource (UniProt) databases [70,71]. The RepeatMasker program screens nucleotide sequences against a library of repetitive elements such as interspersed repeats and low complexity DNA sequences. We discarded sequences with more than 10% repetitive sequence.

Chromosomal localizations in the released chicken genome [11] were reinvestigated using the BLAST-like Alignment Tool (BLAT) [72] and the University of California Santa Clara (UCSC) Genome Browser [73]. Designating functionality to chicken gene products is complicated by the limited knowledge of the avian genome or proteome. Assuming that well conserved protein regions and domains reflect similar protein functionality, cross-species comparisons of several gene products (see Additional Files 5, 6, 7) were done using data from the USCS genome browser, the Ensembl genome browser [74], TIGR chicken gene indices [75], the Pfam [76] and UniProt databases in combination with the BLAST2 (default parameters) [77]. The figures in Additional Files 4 and 5 show sequence similarity and Pfam domains for several of the gene products mentioned in the discussion. Pfam is a database of multiple alignments of protein domains or conserved protein regions [78]. The thicker boxes represent Pfam A domains which are based on manually crafted accurate multiple protein alignments, whereas the multiple colored thinner boxes represent Pfam B domains based on an automatic clustering of a nonredundant protein database.

### Real-time PCR

To confirm the identity as well as the differential expression of selected genes, samples derived from five individual embryos of each sex (randomly selected among the eight that had been pooled for microarray analysis) on ed4 (and, for some genes, ed8) were subjected to real-time PCR. Primers were designed with Primer Express software (Applied Biosystems, Palo Alto, CA, USA), using default settings for the TaqMan mode, and ordered from DNA Technology A/S (Aarhus, Denmark). Primers were designed for amplicons to span exon-exon borders when possible, as determined by the alignment of EST sequences against the chicken genome (see above). Primer sequences are given in Table 6. Primers amplifying the endogenous reference 18S ribosomal RNA were from TaqMan^® ^Ribosomal RNA Control Reagents (Applied Biosystems). For PCR, 2 μg total RNA was reverse transcribed in a final volume of 100 μl using TaqMan Reverse Transcription Reagents (Applied Biosystems) with random hexamer primers according to manufacturer instructions. Reactions excluding MultiScribe Reverse Transcriptase (Applied Biosystems) were performed as negative controls. cDNA targets at a 100-fold (for target genes) or 10,000-fold (for endogenous reference) final dilution were amplified in replicate wells (four for target genes and six for endogenous reference), using primer concentration 250 nM (reverse and forward primer) for target genes and 50 nM for 18S, respectively, in 1× qPCR Mastermix Plus for SYBR Green I (Eurogentec, Seraing, Belgium) in an ABI Prism 7000 Sequence Detector System (Applied Biosystems) with the following thermal profile: 50°C for 2 min, 95°C for 10 min, followed by 40 cycles of 15 sec at 95°C and 1 min at 60°C in a volume of 25 μl. PCR products were checked by monitoring melting curves. Standard curves for each gene were obtained by amplifying (in quadruplicate) a four-fold dilution series of 1:50 through 1:12,800 final volume of a reference mixture containing equal amounts of cDNA from all ten individual samples. For each gene, after removing one outlying value (leaving three values for target genes and five for endogenous reference), a mean normalized gene expression (MNE) was calculated according to Muller *et al *[79]:

MNE=(Etarg⁡et)CTtarg⁡et,well1CTtarg⁡et,well2CTtarg⁡et,well33(Eref)CTref,well1CTref,well2CTref,well33=(Etarg⁡et)CTtarg⁡et,mean(Eref)CTref,mean
 MathType@MTEF@5@5@+=feaafiart1ev1aaatCvAUfKttLearuWrP9MDH5MBPbIqV92AaeXatLxBI9gBaebbnrfifHhDYfgasaacH8akY=wiFfYdH8Gipec8Eeeu0xXdbba9frFj0=OqFfea0dXdd9vqai=hGuQ8kuc9pgc9s8qqaq=dirpe0xb9q8qiLsFr0=vr0=vr0dc8meaabaqaciaacaGaaeqabaqabeGadaaakeaacqWGnbqtcqWGobGtcqWGfbqrcqGH9aqpdaWcaaqaamaabmaabaGaemyrau0aaSbaaSqaaiabdsha0jGbcggaHjabckhaYjabcEgaNjabdwgaLjabdsha0bqabaaakiaawIcacaGLPaaadaWcaaqaaiabdoeadjabdsfaunaaBaaaleaacqWG0baDcyGGHbqycqGGYbGCcqGGNbWzcqWGLbqzcqWG0baDcqGGSaalcqWG3bWDcqWGLbqzcqWGSbaBcqWGSbaBcqaIXaqmaeqaaOGaem4qamKaemivaq1aaSbaaSqaaiabdsha0jGbcggaHjabckhaYjabcEgaNjabdwgaLjabdsha0jabcYcaSiabdEha3jabdwgaLjabdYgaSjabdYgaSjabikdaYaqabaGccqWGdbWqcqWGubavdaWgaaWcbaGaemiDaqNagiyyaeMaeiOCaiNaei4zaCMaemyzauMaemiDaqNaeiilaWIaem4DaCNaemyzauMaemiBaWMaemiBaWMaeG4mamdabeaaaOqaaiabiodaZaaaaeaadaqadaqaaiabdweafnaaBaaaleaacqWGYbGCcqWGLbqzcqWGMbGzaeqaaaGccaGLOaGaayzkaaWaaSaaaeaacqWGdbWqcqWGubavdaWgaaWcbaGaemOCaiNaemyzauMaemOzayMaeiilaWIaem4DaCNaemyzauMaemiBaWMaemiBaWMaeGymaedabeaakiabdoeadjabdsfaunaaBaaaleaacqWGYbGCcqWGLbqzcqWGMbGzcqGGSaalcqWG3bWDcqWGLbqzcqWGSbaBcqWGSbaBcqaIYaGmaeqaaOGaem4qamKaemivaq1aaSbaaSqaaiabdkhaYjabdwgaLjabdAgaMjabcYcaSiabdEha3jabdwgaLjabdYgaSjabdYgaSjabiodaZaqabaaakeaacqaIZaWmaaaaaiabg2da9maalaaabaWaaeWaaeaacqWGfbqrdaWgaaWcbaGaemiDaqNagiyyaeMaeiOCaiNaei4zaCMaemyzauMaemiDaqhabeaaaOGaayjkaiaawMcaamaaCaaaleqabaGaem4qamKaemivaq1aaSbaaWqaaiabdsha0jGbcggaHjabckhaYjabcEgaNjabdwgaLjabdsha0jabcYcaSiabd2gaTjabdwgaLjabdggaHjabd6gaUbqabaaaaaGcbaWaaeWaaeaacqWGfbqrdaWgaaWcbaGaemOCaiNaemyzauMaemOzaygabeaaaOGaayjkaiaawMcaamaaCaaaleqabaGaem4qamKaemivaq1aaSbaaWqaaiabdkhaYjabdwgaLjabdAgaMjabcYcaSiabd2gaTjabdwgaLjabdggaHjabd6gaUbqabaaaaaaaaaa@D655@

E_target _and E_ref _denote the efficiency, and CT_target _and CT_ref _the threshold cycle (CT) of the target and reference gene, respectively, in the PCR amplification. PCR data for each gene are reported in Tables 3, 4, 5 (also see Additional File 3). An un-paired Student's *t*-test, using the MNE values, was used to determine a statistically significant difference between the genders (p < 0.05) for each gene. The comparative C_t _method [80] was used to calculate the average relative log2 fold difference between males and females for each gene.

## List of abbreviations

ABWT, Avian brain W-linked transcript

BLAT, BLAST-like alignment tool

e!, ensemble genome browser

ed, embryonic day

EST, Expressed sequence tag

F, Female

Female genes, genes more highly expressed in females

CAK, Cyclin-dependent kinase activating kinase

Gg, Gallus gallus

HIT, histidine triad motif

Hs, Homo sapiens

M, Male

Male genes, genes more highly expressed in males

MHM, Male hypermethylated

Mm, Mus musculus

MNE, Mean normalized expression

Sc, Saccharomyces

TFIIH, Transcription factor II H

UCSC, University of California Santa Cruz

Xl, Xenopus leavis

Xt, Xenopus tropicalis

## Authors' contributions

All authors conceived and designed the experiments, but KK designed the microarray study. AM performed the experiment related to the animal material. KK, BS and non-authors (see Acknowledgments) carried out the microarray and PCR analysis. BS and KK analyzed the data. BB and LD contributed reagents, materials, and analysis tools. BS, KK and MS drafted the manuscript with significant contributions from all authors. All authors read and approved the final manuscript.

## Supplementary Material

Additional File 1 Expression profile for microarray genes. Expression profile for microarray genes more highly expressed in males according to the chromosomal localization. Click here for file

Additional File 2 Differentially expressed clones. Excel sheet showing annotations and expression data for all 146 differentially expressed clones. Click here for file

Additional File 3 Graphs of PCR results. The average mean normalized expression (MNE) for females (red) and males (blue) of selected gene (listed alphabetically) on ed4 and/or ed8, analyzed by real-time PCR (n = 5). Error bars indicate standard error of the mean. For calculations, see Methods. Click here for file

Additional File 4 Alignment of ABWT. **A**. UCSC genome browser window showing the alignment of ABWT and three additional annotation tracks. 'Non-Chicken RefSeq Genes' shows the alignment of non-chicken RefSeq sequences for *Ube2r2 *from mouse (*Mus musculus*) and zebrafish (*Danio rerio*). 'Chicken mRNAs from GenBank' describes the alignment of additional chicken mRNAs in this region. 'Repeating Elements by RepeatMasker ' shows the parts of the genome region masked for repetitive sequences. This UCSC Genome Browser window can be accessed by searching with 'chrW_random:175,450-180,581' on the UCSC chicken genome browser site [19]. **B**. Alignment data for ABWT against the chicken genome. Click here for file

Additional File 5 Species comparisons of the UBE2R2 protein. Species comparison between chicken (Gg), human (Hs), mouse (Mm), Xenopus leavis/tropicalis (Xl/Xt) and Saccharomyces cerevisiae (Sc) of the UBE2R2 protein using the protein sequences listed in additional file 7. A comparison between Pfam domains (representing protein domains or conserved protein regions) and the number of positive amino acid matches against the chicken sequence is shown (see Methods). Click here for file

Additional File 6 Species comparisons of proteins associated with TFIIH complex. Species comparison between chicken (Gg), human (Hs), mouse (Mm), Xenopus leavis/tropicalis (Xl/Xt) and Saccharomyces cerevisiae (Sc) of TFIIH proteins using the protein sequences listed in additional file 7. A comparison between Pfam domains (representing protein domains or conserved protein regions) and the number of positive amino acid matches against the chicken sequence is shown (see Methods). Click here for file

Additional File 7 Species comparisons of proteins associated with TFIIH complex. List of the protein identities used in Additional Files 5 and 6. Click here for file

## Figures and Tables

**Table 1:** Chromosomal distribution of differentially expressed clones and genes

	Differentially expressed clones and genes					
	'Male'^a^		'Female'^a^		'Total'	

Chromosome	Clones	Genes^b^	Clones	Genes^b^	Clones/Genes^b^	Array clones^c^

Chr W	0	0	9	3	9/3	11
Chr Z	61	56	1	1	62/57	257
Autosomal Chr	29	29	11	11	40/40	10312
Unknown	31	29	4	4	35/33	3327
Total	121	114	25	19	146/133	13907

^a ^'Male' clones/genes are those more highly expressed in males and 'female' clones/genes are more highly or only expressed in females.

^b ^The 146 clones correspond to 134 genes.

^c ^The total chromosomal distribution of clones spotted onto the arrays as determined by a combination of manual and automated annotation.

**Table 2:** Differentially expressed clones located to sex chromosomes

Gene	Chr^a^	More highly expressed in	Clone ID^b^	Log 2 female/male (min-max)^c^	Note
ABWT	W	Female	WLA084D05	1.79–3.3	Confirmed (table 3)
SPINW	W	Female	RJA067D01	0.49–0.55	Confirmed (table 3)
WPKCI-8	W	Female	RJA012A11	3.35–3.96	Confirmed (table 3)
WPKCI-8	W	Female	RJA017G02	1.44–4.01	
WPKCI-8	W	Female	RJA066A02	0.30–1.99	
WPKCI-8	W	Female	RJA081B07	1.35–3.15	
WPKCI-8	W	Female	WLA034C12	1.66–2.77	
WPKCI-8	W	Female	WLA081D02	2.40–3.40	
WPKCI-8	W	Female	WLA096H02	1.85–3.38	
3010001K23RIK	Z	Male	ChEST386O13	-(0.61–0.84)	
AGTPBP1	Z	Male	WLB113E12	-(0.17–0.51)	
AP3S1	Z	Male	RJB097F02	-(0.31–0.58)	
AP3S1	Z	Male	WLB083F12	-(0.34–0.59)	
APG12L	Z	Male	WLB057D05	-(0.12–0.49)	
APTX	Z	Male	RJB091E05	-(0.50–0.59)	
BRT1	Z	Male	RJA090E06	-(0.15–0.28)	
BTF2-P44	Z	Male	WLB041B10	-(0.32–0.52)	Confirmed (table 4)
CAG31437	Z	Male	WLA074E07	-(0.18–0.50)	
CBWD2	Z	Male	RJA111F05	-(0.12–0.55)	
CCDC2	Z	Male	RJB037B08	-(0.42–0.74)	
CCDC2	Z	Male	WLB116D01	-(0.12–0.55)	
CCNH	Z	Male	WLB057D11	-(0.44–0.75)	Confirmed (table 4)
CLA	Z	Male	WLB050E07	-(0.58–0.80)	Confirmed (table 4)
CLA	Z	Male	WLB040F04	-(0.31–0.71)	Confirmed (table 4)
COX7C	Z	Male	RJB015D04	-(0.65–0.79)	
DHFR	Z	Male	RJA087D10	-(0.19–0.72)	Confirmed (table 4)
DNAJA1	Z	Male	WLA058H10	-(0.27–0.45)	
DNAJA1	Z	Male	WLA019B09	-(0.17–0.34)	
EDG2	Z	Male	ChEST21M3	-(0.19–0.37)	
EST unknown	Z	Male	RJA039B04	-(0.39–0.65)	
EST unknown	Z	Male	RJA096G01	-(0.37–0.41)	
EST unknown	Z	Male	RJA118E07	-(0.24–0.50)	
EST unknown	Z	Male	RJB015F01	-(0.19–0.44)	
EST unknown	Z	Male	RJB064G03	-(0.37–0.55)	
EST unknown	Z	Male	WLA033F09	-(0.63–0.76)	
EST unknown	Z	Male	WLA075A02	-(0.31–0.42)	
EST unknown	Z	Male	WLB060B10	-(0.38–0.50)	
EST unknown	Z	Male	WLB117F04	-(0.13–0.40)	
FLJ21657	Z	Male	WLB055E01	-(0.27–0.44)	
FNTA	Z	Male	ChEST249K9	-(0.13–0.43)	
GK004	Z	Male	RJB071E12	-(0.30–0.42)	
GNG10	Z	Male	WLB054D11	-(0.51–0.62)	
HMGCOA	Z	Male	ChEST873P23	-(0.32–0.63)	Confirmed (table 4)
HNRNPK	Z	Male	WLB066G09	-(0.15–0.39)	
HSPC097	Z	Male	WLB040G10	-(0.15–0.35)	
IDUA	Z	Male	WLA061C06	-(0.23–0.45)	
IKAP	Z	Male	WLB112G04	-(0.62–0.75)	
LOC389293	Z	Male	RJA119B07	-(0.16–0.36)	
LOC427191	Z	Male	WLB133D02	-(0.43–0.49)	
LOC427252	Z	Male	WLB033H03	-(0.23–0.35)	
MAK10	Z	Male	RJA024F07	-(0.51–0.77)	
MHM region	Z	Female	RJA001B07	0.44–2.58	Confirmed (table 4)
MSH3	Z	Male	ChEST701C3	-(0.57–0.90)	
NANS	Z	Male	WLB031E11	-(0.23–0.29)	
PAM	Z	Male	RJB064A12	-(0.15–0.39)	
PAM	Z	Male	WLA096C08	-(0.35–0.47)	
PAR-3	Z	Male	ChEST812E15	-(0.47–0.59)	
PG-M	Z	Male	ChEST376N5	-(0.18–0.41)	
PIP5K1B	Z	Male	WLA025H03	-(0.16–0.37)	
PLAA^d^	Z	Male	RJB016B03	-(0.60–0.76)	
RAD1	Z	Male	ChEST963P4	-(0.45–0.66)	
RAD17	Z	Male	RJA010B11	-(0.18–0.38)	
RASA1	Z	Male	ChEST57017	-(0.58–0.70)	
RPS6	Z	Male	WLB048H08	-(0.45–0.70)	
RPS6^e^	Z	Male	WLB065B04	-(0.47–0.67)	
RPS23	Z	Male	WLA053B06	-(0.67–0.80)	
SMAD2	Z	Male	WLA084F02	-(0.32–0.42)	Confirmed (table 4)
SOLT	Z	Male	ChEST368P13	-(0.49–0.71)	Confirmed (table 4)
SPINZ	Z	Male	ChEST720C2	-(0.32–0.44)	Confirmed (table 4)
TINP1	Z	Male	WLB058A04	-(0.64–0.74)	Confirmed (table 4)
TLE4	Z	Male	RJA118C01	-(0.11–0.39)	
VCP	Z	Male	WLA025D11	-(0.13–0.28)	
ZNT-5	Z	Male	WLB088E10	-(0.41–0.56)	

^a ^Chromosome

^b ^Can be used to search against GenBank

^c ^Log_2 _female/male (min – max) lists the range of average expression differences between females and males between ed4 and ed10.

^d ^Original annotation of spotted clone was RJB020E03, which codes for autosomal ACAD8.

^e ^Original annotation of spotted clone was WLB065A04, which codes for autosomal AATF.

**Table 3:** W-linked genes

	Log2 female/male						
		
	ed4^a^			ed8^a^			
			
Gene	Array^b^	PCR^b^	p-value	Array^b^	PCR^b^	p-value	Note
ABWT^c^	1.78	8.73 ∞	p < 0.05	2.87	NA^f^	NA^f^	
SPINW	0.27	11.50 ∞	p < 0.05	0.49	NA^f^	NA^f^	
UBE2R2	NS^e^	NDE^d^	-	NS^e^	NA^f^	NA^f^	
WPKCI-8	4.05	13.36 ∞	p < 0.05	1.58	NA^f^	NA^f^	

^a ^Embryonal day 4/8.

^b ^Array and PCR values are given as log2 female/male ratio expression differences

^c ^ABWT is a clone expressed in females and is located adjacent to the UBE2R2 gene.

^d ^No differential expression

^e ^Not spotted on the microarray.

^f ^Not available

∞ Expressed only in females

**Table 4:** Z-linked genes

	Log2 female/male						
		
	ed4^a^			ed8^a^			
			
Gene	Array^b^	PCR^b^	p-value	Array^b^	PCR^b^	p-value	Note
ANXA1^c^	NDE^e^	NDE^e^	-	NDE^e^	NDE^e^		
BTF2-P44	-0.26	-0.44	0.06	-0.22	-0.73	p < 0.05	
CCNH	-0.71	-1.09	p < 0.05	-0.44	NA^g^		
CDK7	NS^f^	-0.54	0.12	NS^f^	-0.67	p < 0.05	
CLA	-0.46	-1.12	p < 0.05	-0.28	NA^g^		
DHFR	-0.71	-1.00	p < 0.05	-0.56	NA^g^		
DMRT1	NA^g^	NDE^e^	-	NDE^e^	-		
HINT	NS^f^	-1.17	p < 0.05	NS^f^	-1.35	p < 0.05	
HMGCOA	-0.37	-0.68	p < 0.05	-0.32	NA^g^		
SMAD2	-0.32	-0.8	p < 0.05	-0.42	NA^g^		
SOLT	-0.71	-0.95	p < 0.05	-0.49	NA^g^		
SPINZ	-0.32	-0.8	p < 0.05	-0.21	-1.35	p < 0.05	
TINP1	-0.63	-0.93	p < 0.05	-0.74	NA^g^		
MHM array clone^d^	2.57	8.21	p < 0.05	0.44	NA^g^		
MHM GenBank^d^	NS^f^	7.73	p < 0.05	NS^f^	NA^g^		
TXN^c^	0	1.05	p < 0.05	NA^g^	NA^g^		Contaminated by vector sequence
ZO-2^c^	NDE^e^	NDE^e^	-	NDE^e^	NA^g^		
ZOV3^c^	NDE^e^	NDE^e^	-	NDE^e^	NA^g^		

^a ^Embryonal day 4/8.

^b ^Array and PCR values are given as log2 female/male ratio expression differences

^c ^ANXA1 (*Annexin 1*), TXN (*Thioredoxin*), *ZO-2 *and *ZOV3 *were randomly chosen as not differentially expressed Z-linked genes.

^d ^Two MHM transcripts are listed; *RJA001B07 *(spotted on the array) and *AB046698 *(GenBank).

^e ^No differential expression

^f ^Not spotted on the microarray.

^g ^Not available

**Table 5:** Autosomal genes and genes having an unknown location

			Log 2 female/male						
				
			ed4^a^			ed8^a^			
					
Gene	Agreement between array and PCR data	Chr^b^	Array^c^	PCR^c^	p-value	Array^c^	PCR^c^	p-value	Notes
ACAA2	Yes	U^d^	-0.50	-0.80	p < 0.05	-0.18	-1.00	p < 0.05	
CETN3	Yes	U^d^	-0.66	-0.97	p < 0.05	-0.54	-1.29	p < 0.05	
MAT1	Yes	5	NDE^e^	NDE^e^	-	NDE^e^	NDE^e^	-	
P311	Yes	10	-0.77	-0.97	p < 0.05	-0.62	-0.92	p < 0.05	
AATF	No	19	-0.53	NDE^e^	-	-0.67	NA^f^		Real identity is RPS6 from Chr Z
ACAD8	No	24	-0.72	NDE^e^	-	-0.74	NA^f^		Real identity is PLAA from Chr Z
CAT-1	No	4	0.97	NDE^e^	-	0.39	NA^f^		
CEZANNE2	No	10	5.00	NDE^e^	-	1.95	NA^f^		
CX3CR	No	2	-0.67	0.34	-	-0.49	NA^f^		
HERP1/HEY2	No	3	-0.49	-0.23	-	-0.58	NA^f^		Real identity is ChEST696L1 from Chr 12
MPRL3	No	2	0.49	-0.38	-	0.72	NA^f^		Real identity is WLB075E02 (Rep. seq) from unknown chr. location^g^
NAT5	No	7	1.00	-0.27	-	0.74	NA^f^		
PRTD-NY3	No	28	0.67	-0.29	-	0.42	NA^f^		Real identity is RJB067C04 from unknown chr. location^g^
RJA094A01	No	9	0.70	NDE^e^	-	0.45	NA^f^		
ZNF216	No	U^d^	0.32	0.15	-	0.41	NA^f^		

^a ^Embryonal day 4/8.

^b ^Chromosome

^c ^Array and PCR values are given as log2 female/male ratio expression differences

^d ^Unknown chromosomal localization

^e ^No differential expression

^f ^Not available

^g ^Rep. seq stands for repetitive sequence.

**Table 6:** Primer sequences

Gene	Amplicon (nt)	Forward (5'-3')	Reverse (3'-5')
AATF	64	TTACCGCTCACTGTTTGGAAGA	TCCCGGATGCCAACCA
ACAA2	76	TCTGGTTTCCAGTCCATTGCT	CTCCACCACACAGAACCACTTC
ACAD8	70	CATGGCCAAGCTGTTTGCTA	GCCCCCGTGCATCTGTAG
ANXA1	78	TGAATGATGATCTTGCTGACAATG	CGTTAACATCCGTCCCTTTCC
BTF2-P44	85	CCTCCACCTGCCAGTTCAAC	CCTGGTCAGAAAGGGAAGCA
CAT1	71	CACGCTGATGGTGCCCTACT	AGCCCACCGCCTTGAAAG
CCNH	73	AACCGAAAGTTTCGCAGCAA	GGGCCTCCAGCAGGAAAG
CDK7	78	GGGACAGATCGTGGCTATCAAA	TGAGAGCTGTTCTGTTGATTCCA
CETN3	77	GAAATACTCAAGGCGTTCAAATTGT	GCAACCCGACGCAGGTT
CEZANNE2	67	CCACAACTGGTGATGGAAACTG	CGGTCATGAAATCCCCACAT
CEZANNE2 e!	69	GGCCACAACTGGTGATGGA	CGGTCATGAAATCCCCACAT
CLA	51	GTAAGTGGAGAGAGGAGCAAAAGG	AGTTTGCATCAAGCTGCTCAAG
CX3CR	74	CACCGTGTCTGCTTCAAGTACTG	TATGTGCGGACGTCATAATGATT
DHFR	79	ATGGGCATCGGGAAGGA	TGCTGGTCATTCTCTGGAAGTACT
DMRT1	81	GAAGCCTCCCAGCAACATACA	CATGGCATGGCGGTTCTC
HERP1/HEY2	68	GCGACCGGAGGTAAAGGTTATT	CGGAAGCCAATGCTCATAAAA
HINT	77	ATATTCTGGGAGGTCGTCAGTTG	TTGTACACATGCAGCATCTCTTGT
HMGCOA	84	GAAGAAAATAAACCAAACCCTGTAACA	GCGACTGTGTGCATGAACAAG
MAT1	62	CCCCTCCCTGAAGCTGATG	GCTCCACGCAGCTCTCACA
MHM array clone	76	CAACAGCACTACTGGAGCCATATG	GGAAGCCCCTGCAAAGAAG
MHM GenBank	69	GAAATGCCTCGCCCTTTAAGT	TGACTGCCCTTGGTTGTCTGT
MRPL3	72	GAGTTGGTGTTGTTGCGGTAA	TGACAGCGTGCTTCTTTCCT
NAT5	51	CTGACGGAGACCTACGGCAT	CCAGTGTGCCAGGTACTGCA
P311	75	TCATGGTTTCCTTGTGGTAAGTGA	TCCAAAATAGCACCATTCATCAGT
PRTD-NY3	68	TCACACGGTCTCCATACCTCAA	CCGACAGCCTGCCATCTC
RJA094A01	74	GGGAGGGTGGATCTGTGACA	ACAGCCCTTCCCGTAAAAGAG
SMAD2	51	AGTGGGACACAACAGGCCTT	CGAGAGATCTGGTTTGTTCAGAGAA
SOLT	61	TTATTAGGAGAAGAAGAGTTGCAGAAAG	CTGCTGACAGCACCATTTCAA
SPINW	102	CTGTTCAAGAATAAATAGAATCACAATGG	TTCAGTCCTCACATAGTCCAACTTAGTT
SPINZ	101	CCAAGTTCCTGTAAATCCCTCTCTT	CAAGCGCAGAAACTCTTTCATCT
TINP1	77	AAGCTGGGAAATGGGAGGTT	AAGCTGGGAAATGGGAGGTT
TXN	72	TGATGTGGTGTTCATTGAAATTGA	GGCATGCACTTCACATCACAGT
UBE2R2	76	CCAAAGCTGAGGCAGAAAAGG	GGCACTTTAGTTTTGATGCAGTACTC
WLA084D05/ABWT	72	TGGGATGGCTGCATGTGTA	AACCCCGCCACTGGTATTC
WPKCI-8	51	AGATTGTGGCGCACCTCTTC	CACTTCTCGCCAACAATCATCA
ZNF216	67	GCCCCGGCCCCATT	GCATTAAAACTACAGAATCCTCTTTGC
ZO-2	83	CCTTTTCTTCCCCTTTTATTAATGCT	TCTGTTGCATCAAGTTTATCTCCTAGA
ZOV3	86	CGATACCTGCCTCCAGTGTGT	GAGGAACAGACCTATTTACAAACTGAAA
