# Supplementary figures and images for: Sex-dependent gene expression in early brain development of chicken embryos
Source: BMC Neurosci. 2006 Feb 15;7:12. doi: 10.1186/1471-2202-7-12 (PMC1386693; doi:10.1186/1471-2202-7-12)

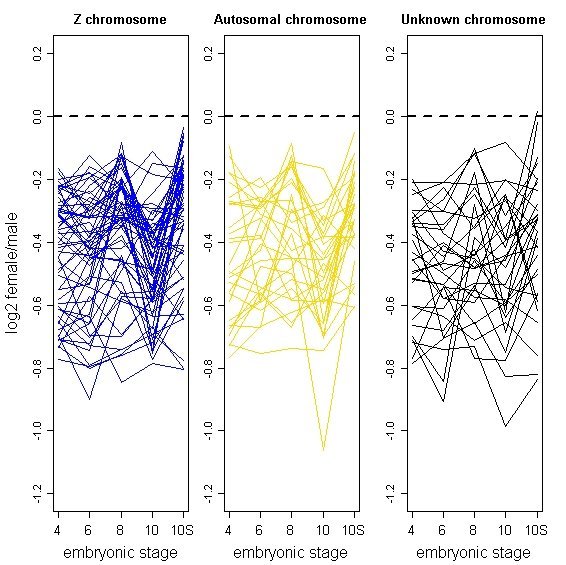

Supplement: Additional File 1 — Expression profile for microarray genes. Expression profile for microarray genes more highly expressed in males according to the chromosomal localization. [file 1471-2202-7-12-S1.jpeg]

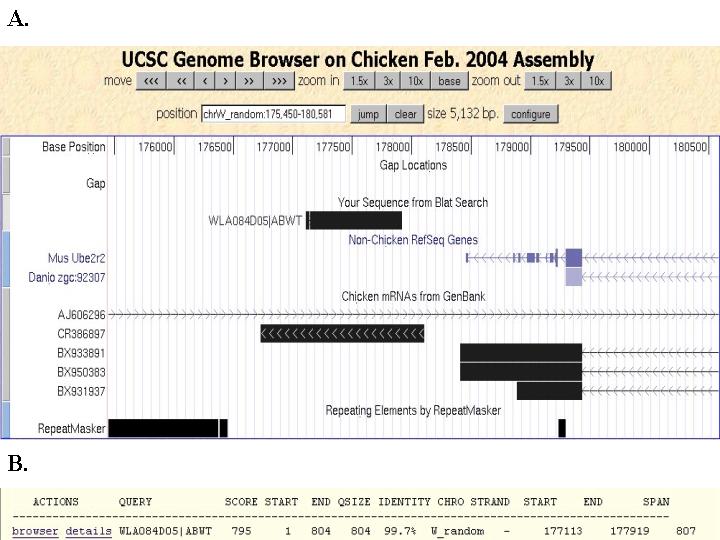

Supplement: Additional File 4 — Alignment of ABWT. A. UCSC genome browser window showing the alignment of ABWT and three additional annotation tracks. 'Non-Chicken RefSeq Genes' shows the alignment of non-chicken RefSeq sequences for Ube2r2 from mouse (Mus musculus) and zebrafish (Danio rerio). 'Chicken mRNAs from GenBank' describes the alignment of additional chicken mRNAs in this region. 'Repeating Elements by RepeatMasker ' shows the parts of the genome region masked for repetitive sequences. This UCSC Genome Browser window can be accessed by searching with 'chrW_random:175,450-180,581' on the UCSC chicken genome browser site [19]. B. Alignment data for ABWT against the chicken genome. [file 1471-2202-7-12-S4.jpeg]

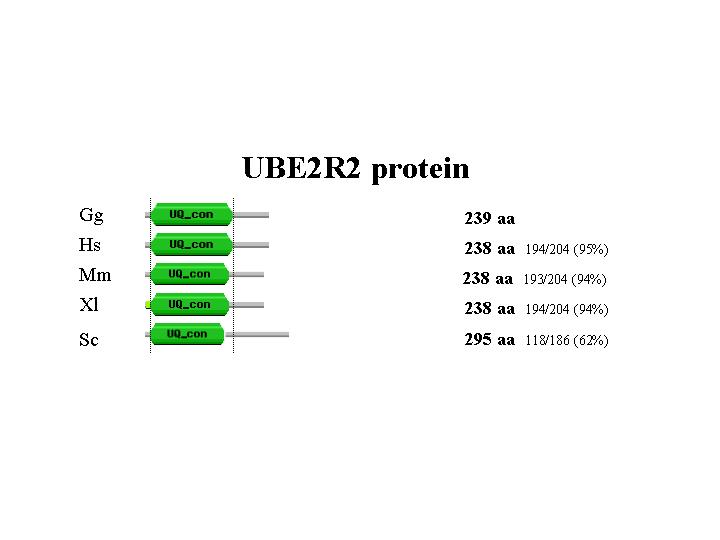

Supplement: Additional File 5 — Species comparisons of the UBE2R2 protein. Species comparison between chicken (Gg), human (Hs), mouse (Mm), Xenopus leavis/tropicalis (Xl/Xt) and Saccharomyces cerevisiae (Sc) of the UBE2R2 protein using the protein sequences listed in additional file 7. A comparison between Pfam domains (representing protein domains or conserved protein regions) and the number of positive amino acid matches against the chicken sequence is shown (see Methods). [file 1471-2202-7-12-S5.doc]

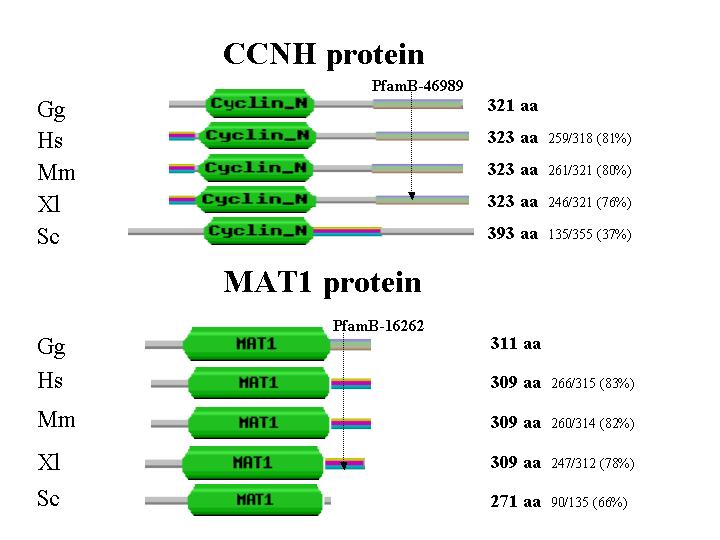


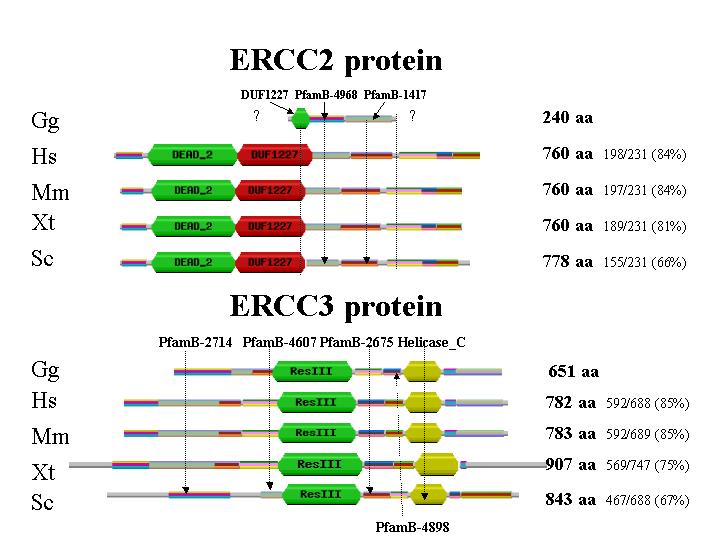


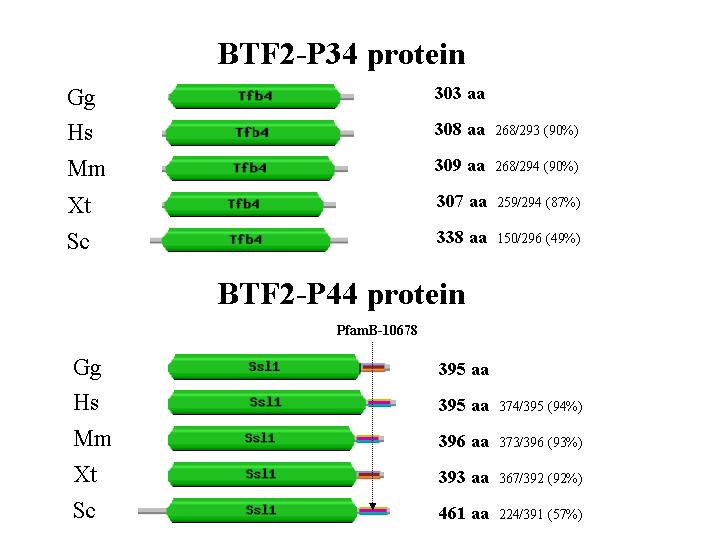


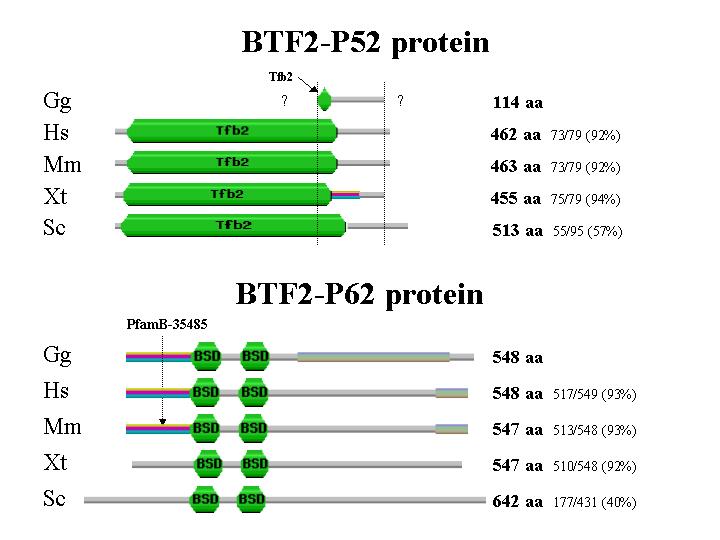

Supplement: Additional File 6 — Species comparisons of proteins associated with TFIIH complex. Species comparison between chicken (Gg), human (Hs), mouse (Mm), Xenopus leavis/tropicalis (Xl/Xt) and Saccharomyces cerevisiae (Sc) of TFIIH proteins using the protein sequences listed in additional file 7. A comparison between Pfam domains (representing protein domains or conserved protein regions) and the number of positive amino acid matches against the chicken sequence is shown (see Methods). [file 1471-2202-7-12-S6.doc]
