# Supplementary material for: Sex-dependent gene expression in early brain development of chicken embryos
Source: BMC Neurosci. 2006 Feb 15;7:12. doi: 10.1186/1471-2202-7-12 (PMC1386693; doi:10.1186/1471-2202-7-12)
Supplement: Additional File 7 — Species comparisons of proteins associated with TFIIH complex. List of the protein identities used in Additional Files 5 and 6. [file 1471-2202-7-12-S7.doc]

|  |  |  |  |  |  |  |
| --- | --- | --- | --- | --- | --- | --- |
| **Sub-complex** | **Name** | **Chicken** | **Human** | **Mouse** | **Xenopus** | **Yeast** |
| - | UBE2R2 | Q5ZHR6_CHICK | Q712K3_HUMAN | Q8VDE5_MOUSE | Q76EZ3_XENLA | UBC3_YEAST |
| CAK | CDK7 | ChEST749g14 (frame +2) | CDK7_HUMAN | CDK7_MOUSE | CDK7_XENLA | KIN28_YEAST |
| CAK | CCNH | ENSGALT00000025216.1 | CCNH_HUMAN | CCNH_MOUSE | CCNH_XENLA | CCL1_YEAST |
| CAK | MAT1 | ENSGALT00000019434.1 | MAT1_HUMAN | MAT1_MOUSE | MAT1_XENLA | P89104_YEAST |
| Core | ERCC2 * | TC226675 (frame +2) | ERCC2_HUMAN | ERCC2_MOUSE | Q66II2_XENTR | RAD3_YEAST |
| Core | ERCC3 * | ENSGALT00000018803.1 | ERCC3_HUMAN | ERCC3_MOUSE | Scaffold_633.8 | RAD25_YEAST |
| Core | BTF2-P34* | ENSGALT00000005130.1 | TF2H3_HUMAN | TF2H3_MOUSE | TC11047 (frame +2) | Q12004_YEAST |
| Core | BTF2-P44* | ENSGALT00000023876.1 | TF2H2_HUMAN | TF2H2_MOUSE | TC2654 (frame +1) | SSL1_YEAST |
| Core | BTF2-P52* | BX267456(frame+1) | TF2H4_HUMAN | TF2H4_MOUSE | TC10428 (frame+3) | Q02939_YEAST |
| Core | BTF2-P62 | ENSGALT00000010146.1 | TF2H1_HUMAN | TF2H1_MOUSE | Q6DIF6_XENTR | TFB1_YEAST |
|  |  |  |  |  |  |  |

* The sequences for chicken ERCC2 and BTF2-P52 and several Xenopus genes are retrieved from the TIGR gene indexes (chicken and Xenopus tropicalis). For Xenopus ERCC3 the GeneScan annotation for scaffold 633.8 in the Xenopus 2004 genome assembly is used. The protein sequence for chicken BTF2-P52 is derived from GenBank entry BX267456.
