# Supplementary material for: Sex-dependent gene expression in early brain development of chicken embryos
Source: BMC Neurosci. 2006 Feb 15;7:12. doi: 10.1186/1471-2202-7-12 (PMC1386693; doi:10.1186/1471-2202-7-12)
Supplement: Supplementary file 4 [file 1471-2202-7-12-S3.doc]

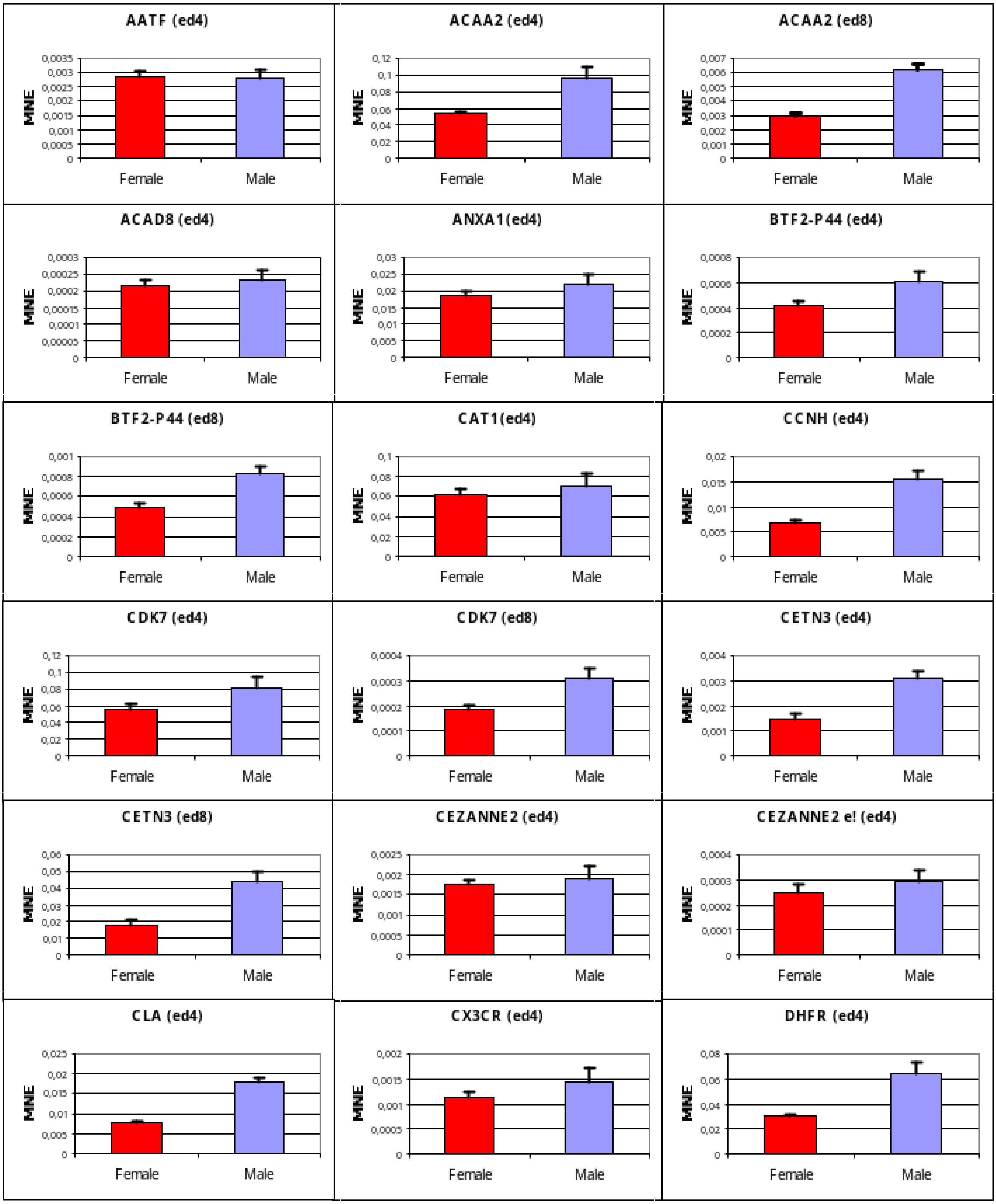
AATF (ed4)
MNE
Female
Male
ACAA2 (ed4)
MNE
Female
Male
ACAA2 (ed8)
MNE
Female
Male
ACAD8 (ed4)
MNE
Female
Male
ANXA1 (ed4)
MNE
Female
Male
BTF2-P44 (ed4)
MNE
Female
Male
BTF2-P44 (ed8)
MNE
Female
Male
CAT1 (ed4)
MNE
Female
Male
CCNH (ed4)
MNE
Female
Male
CDK7 (ed4)
MNE
Female
Male
CDK7 (ed8)
MNE
Female
Male
CETN3 (ed4)
MNE
Female
Male
CETN3 (ed8)
MNE
Female
Male
CEZANNE2 (ed4)
MNE
Female
Male
CEZANNE2 e! (ed4)
MNE
Female
Male
CLA (ed4)
MNE
Female
Male
CX3CR (ed4)
MNE
Female
Male
DHFR (ed4)
MNE
Female
Male

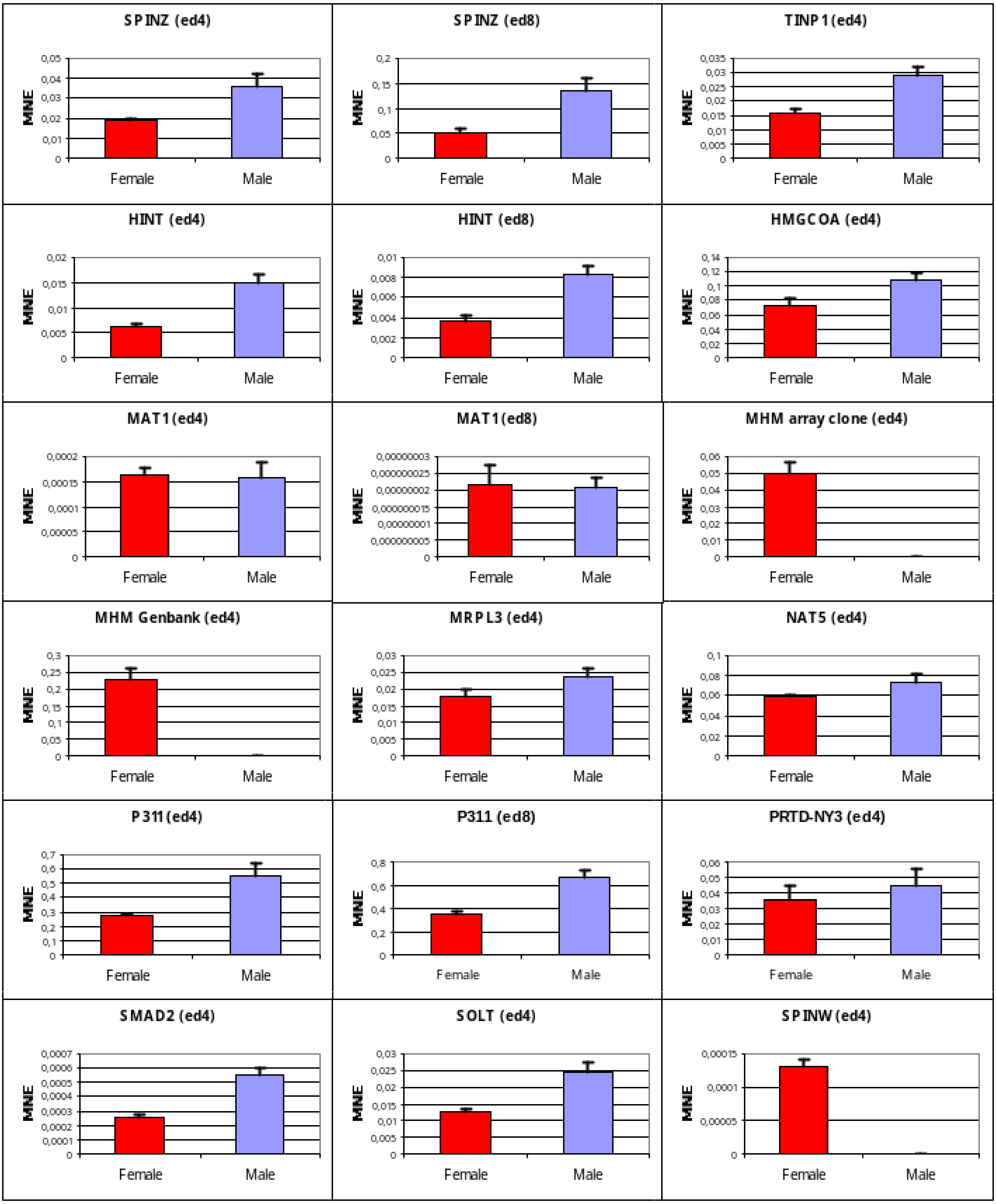
SPINZ (ed4)
0,05
0,04
0,03
0,02
0,01
0
MNE
Female
Male
SPINZ (ed8)
0,2
0,15
0,1
0,05
0
MNE
Female
Male
TINP1 (ed4)
0,035
0,03
0,025
0,02
0,015
0,01
0,005
0
MNE
Female
Male
HINT (ed4)
0,02
0,015
0,01
0,005
0
MNE
Female
Male
HINT (ed8)
0,01
0,008
0,006
0,004
0,002
0
MNE
Female
Male
HMGCOA (ed4)
0,14
0,12
0,1
0,08
0,06
0,04
0,02
0
MNE
Female
Male
MAT1 (ed4)
0,0002
0,00015
0,0001
0,00005
0
MNE
Female
Male
MAT1 (ed8)
0,00000003
0,000000025
0,00000002
0,000000015
0,00000001
0,000000005
0
MNE
Female
Male
MHM array clone (ed4)
0,06
0,05
0,04
0,03
0,02
0,01
0
MNE
Female
Male
MHM Genbank (ed4)
0,3
0,25
0,2
0,15
0,1
0,05
0
MNE
Female
Male
MRPL3 (ed4)
0,03
0,025
0,02
0,015
0,01
0,005
0
MNE
Female
Male
NAT5 (ed4)
0,1
0,08
0,06
0,04
0,02
0
MNE
Female
Male
P311 (ed4)
0,7
0,6
0,5
0,4
0,3
0,2
0,1
0
MNE
Female
Male
P311 (ed8)
0,8
0,6
0,4
0,2
0
MNE
Female
Male
PRTD-NY3 (ed4)
0,06
0,05
0,04
0,03
0,02
0,01
0
MNE
Female
Male
SMAD2 (ed4)
0,0007
0,0006
0,0005
0,0004
0,0003
0,0002
0,0001
0
MNE
Female
Male
SOLT (ed4)
0,03
0,025
0,02
0,015
0,01
0,005
0
MNE
Female
Male
SPINW (ed4)
0,00015
0,0001
0,00005
0
MNE
Female
Male

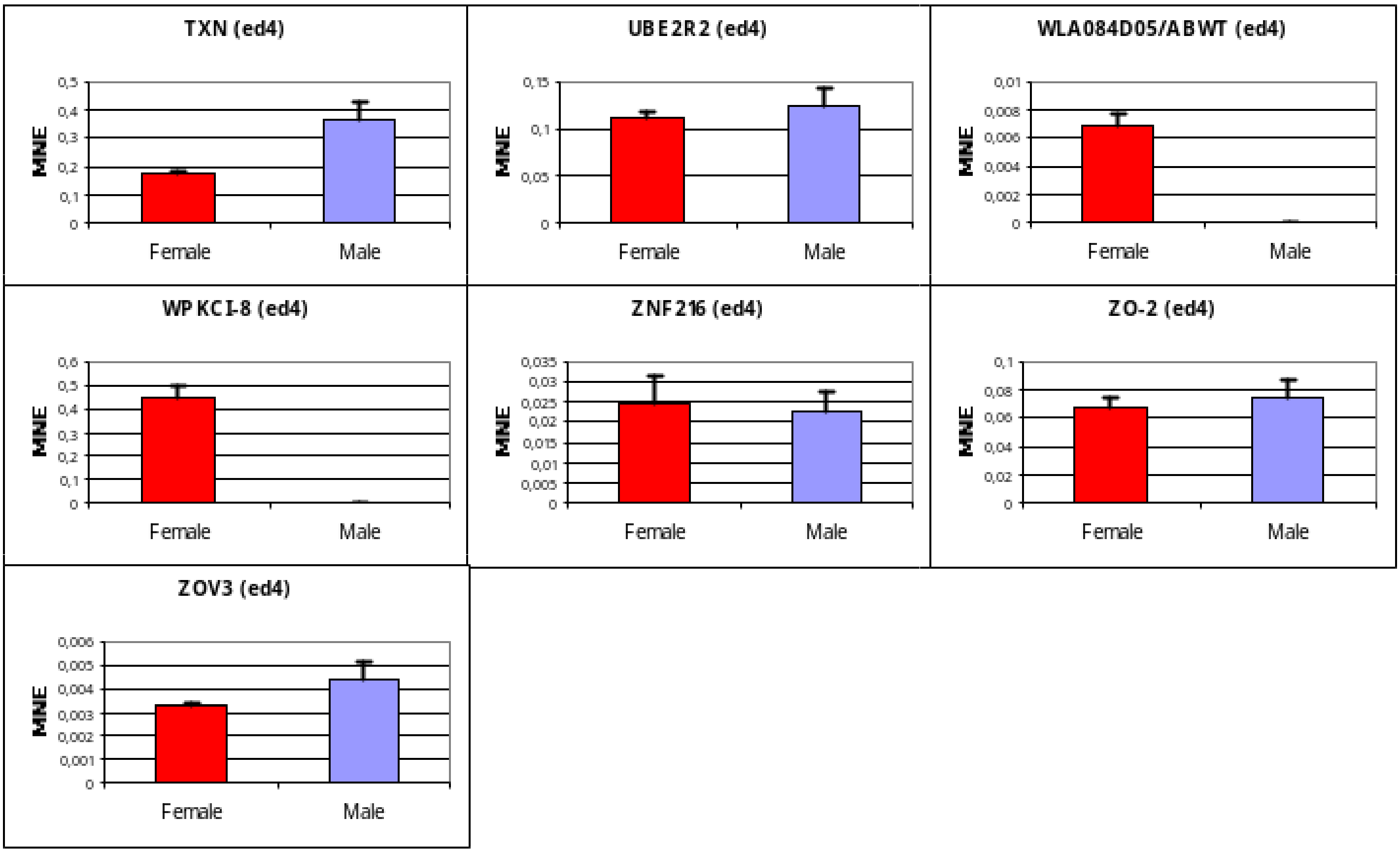
TXN (ed4)
MNE
0,5
0,4
0,3
0,2
0,1
0
Female
Male
UBE2R2 (ed4)
MNE
0,15
0,1
0,05
0
Female
Male
WLA084D05/ABWT (ed4)
MNE
0,01
0,008
0,006
0,004
0,002
0
Female
Male
WPKCI-8 (ed4)
MNE
0,6
0,5
0,4
0,3
0,2
0,1
0
Female
Male
ZNF216 (ed4)
MNE
0,035
0,03
0,025
0,02
0,015
0,01
0,005
0
Female
Male
ZO-2 (ed4)
MNE
0,1
0,08
0,06
0,04
0,02
0
Female
Male
ZOV3 (ed4)
MNE
0,006
0,005
0,004
0,003
0,002
0,001
0
Female
Male
